# A Syllabus of Lectures on Odontology

**Published:** 1884-12

**Authors:** C. L. Ford

**Affiliations:** Professor Anatomy and Physiology in the Dental College of the University of Michigan


### A Syllabus of Lectures on Odontology.

BY C. L. FORD, M. D., D.D.S.

Professor Anatomy and Physiology in the Dental College of the University of Michigan.

This little work was prepared by Professor Ford with primary reference to the class in the Dental College of the University of Michigan. The preface says: "To assist the students to acquire more knowledge of this subject than they are likely otherwise to obtain." The field of Odontology, both human and comparative, is very fully embraced in this Syllabus, and nowhere else can the information here embodied be found, at least in so practicable a form. The author says: "To facilitate still further their study, I have added questions wherein I call attention sharply to such facts and principles as are likely, in many cases, to be overlooked. My aim is to place in the hands of every student the material for study, not simply to read over without thought or purpose, but to make the facts stated a part of his individual knowledge. These questions will enable the student to examine himself repeatedly, and far more effectively and usefully than I can with the time at my command." In order to give an idea of the scope of this Syllabus, we add here a few terms and definitions which should become familiar to every student.

HOMODONT dentition is where the teeth are of a uniform shape, as in some Cetacea.

HETRODONT dentition is where the teeth vary in form, as in a dog or horse.

A MONOPHYODONT is an animal that develops only one single set of teeth, single dentition.

A DIPHYODONT is an animal producing two sets of teeth, as in man, double dentition.

A MULTIPHYODONT is an animal developing new teeth during life, an indefinite number, as in reptiles and fishes. This is multiple dentition.

ODONTOBLASTS, cells on the surface of a tooth pulp as calcification progresses.

OSTEOBLAST, cells on the surface of bone where ossification is progressing.

OSTEOCLASTS, cells on the surface of tissues next to bone or tooth where its absorption occurs.

DIASTEMA, a space between teeth in the dental arch in all mammals except man.

To this syllabus are added questions relating to the teeth. Some of these are the same as have been published before by Professor Ford, but they have been revised, and many new ones added. These, also, are invaluable to the student as aiding him in his study. These are important to the student as leading him up to the particular subjects and points to which he must give attention, study, and investigation. This presentation of questions most thoroughly covers the ground to which it pertains, and he who masters the answers of these questions will have a knowledge of the subject that well be a firm foundation on which he can build his subsequent structure.

Name the different parts of the inferior maxilla.
What part of it joins another bone in articulation?
With what bone does the condyle articulate?
Describe the shape of the glenoid fossa in man.
What structure covers these articulating surfaces?
What elevation in front of the glenoid fossa of man?
Describe the inter-articular cartilage in man.
Describe the form of the glenoid fossa in felidæ.
What is the form of the condyle in the same?
What is the motion of the jaw with such shapes?
In what way do the motions of the sheep's jaw differ?
How do the corresponding bones differ in the ox?
Describe the motions of the jaw in the ruminantia.
What is the form of the glenoid fossa in rodentia?
Compare the condyle of the rodent and the felidæ.
Compare movements of mastication in sheep and dog.
What is the principal use of teeth in fishes?
What motion is required when used for prehension?
What are the motions of the jaw in reptiles?
What are the muscles of mastication in man?
Name the principal action of each muscle.
By what nerve are these muscles supplied?

